# Predictors of Stroke in Patients With Atrial Fibrillation: A Retrospective Study

**DOI:** 10.7759/cureus.104243

**Published:** 2026-02-25

**Authors:** Dalvir Soor, Kanza Majeed, Zahida Batool Bukhari, Areesha Zeeshan, Kyaw Naing Soe, Maha Azmat, Ayesha Saeed

**Affiliations:** 1 Acute Medicine, Basildon University Hospital, Mid and South Essex NHS Foundation Trust, Basildon, GBR; 2 Internal Medicine, Iffat Anwar Medical Complex, Lahore, PAK; 3 Radiology, Combined Military Hospital, Lahore, PAK; 4 Biology, Roots Ivy International, Rawalpindi, PAK; 5 Internal Medicine, Islam Central Hospital, Sialkot, PAK; 6 Cardiology, Sir Ganga Ram Hospital, Lahore, PAK

**Keywords:** anticoagulation, atrial fibrillation, cha₂ds₂-vasc score, hypertension, left atrial enlargement, stroke

## Abstract

Background

Stroke remains one of the most serious complications of atrial fibrillation (AF) and is associated with substantial long-term disability and mortality worldwide.

Objective

The objective of this study is to determine the frequency and independent predictors of ischemic stroke among patients with AF and assess the associations of demographic, clinical, and echocardiographic factors with stroke occurrence.

Methods

This retrospective study was conducted at Sir Ganga Ram Hospital, Lahore, Pakistan, from October 2022 to October 2025 and included 225 patients with non-valvular AF. Medical records were reviewed to collect demographic characteristics, comorbidities, laboratory and echocardiographic findings, and anticoagulation status. Stroke events included both prior history and incident events confirmed by CT or MRI during the study period. “Inadequate anticoagulation” was defined as subtherapeutic INR (<2.0) for warfarin users or documented missed/discontinued doses for direct oral anticoagulant users. Multivariable logistic regression was performed to identify independent predictors of stroke, reporting adjusted odds ratios (aORs), 95% confidence intervals (CIs), and p-values.

Results

Stroke occurred in 78 patients (34.7%). Multivariable analysis identified several independent predictors of stroke. Patients aged 65 years or older had more than twice the odds of stroke (aOR 2.41; 95% CI 1.32-4.39; p = 0.004). Hypertension was also associated with increased risk (aOR 1.98; 95% CI 1.05-3.72; p = 0.03). Echocardiographic findings such as left atrial enlargement (aOR 2.89; 95% CI 1.56-5.36; p = 0.001) and the presence of left atrial appendage (LAA) thrombus or spontaneous echo contrast (SEC) (aOR 3.72; 95% CI 1.72-8.03; p < 0.001) significantly predicted stroke. Inadequate anticoagulation conferred a threefold higher risk (aOR 3.15; 95% CI 1.72-5.77; p < 0.001), and smoking was also an independent predictor (aOR 1.84; 95% CI 1.05-3.23; p = 0.03). Other variables, including diabetes mellitus, dyslipidemia, and reduced left ventricular ejection fraction, were not independently associated with stroke after adjustment.

Conclusion

In this cohort, stroke in AF patients was strongly associated with older age, uncontrolled hypertension, left atrial enlargement, LAA thrombus/SEC, inadequate anticoagulation, and smoking. Early identification of high-risk individuals and optimization of anticoagulation therapy are critical for reducing thromboembolic events. Prospective studies are warranted to validate these findings and guide risk stratification in broader populations.

## Introduction

Atrial fibrillation (AF) is the most prevalent sustained cardiac arrhythmia globally and is a major contributor to ischemic stroke and cardiovascular mortality [1]. Beyond being a disorder of cardiac rhythm, AF promotes blood stasis within the atria, endothelial dysfunction, and thrombus formation, leading to an approximately fivefold increased risk of ischemic stroke compared to individuals in sinus rhythm [2,3]. AF-related strokes are often more disabling and carry higher mortality, reflecting the combined effects of structural atrial remodeling and systemic prothrombotic mechanisms [4].

Thrombus formation in non-valvular AF most commonly occurs in the left atrial appendage (LAA), which accounts for the majority of intracardiac thrombi [5]. Impaired atrial contraction, atrial enlargement, and associated hemodynamic changes further enhance thrombogenesis [6]. For this reason, structural cardiac abnormalities such as left atrial enlargement and the presence of LAA thrombus or spontaneous echo contrast (SEC) are clinically relevant markers of increased thromboembolic risk.

Risk stratification for stroke in AF is commonly performed using validated clinical tools such as the CHA₂DS₂-VASc score, which incorporates age, hypertension, diabetes mellitus, prior stroke, vascular disease, and sex category [7,8]. Although widely recommended, this scoring system was largely derived from Western cohorts, and its predictive performance in South Asian populations remains less clearly defined. Importantly, the score does not directly account for anticoagulation adequacy, medication adherence, or structural cardiac parameters such as left atrial enlargement, which may significantly influence stroke risk in real-world clinical settings.

In Pakistan, AF is frequently encountered in tertiary care centers, often in patients with poorly controlled hypertension, high rates of smoking, and inconsistent long-term follow-up. Warfarin remains commonly prescribed due to cost considerations, and challenges such as subtherapeutic international normalized ratio (INR), irregular monitoring, and medication non-adherence are prevalent. Despite these contextual factors, there is limited local data evaluating how anticoagulation quality, echocardiographic abnormalities, and modifiable behavioral risk factors contribute to stroke occurrence in Pakistani patients with AF.

Most available literature from the region focuses on prevalence or general stroke burden rather than identifying independent predictors within local healthcare constraints [9-13]. Furthermore, few studies from Pakistan have examined the additive value of structural cardiac findings or anticoagulation adequacy beyond traditional clinical risk factors included in the CHA₂DS₂-VASc score. Understanding whether locally relevant factors, such as suboptimal anticoagulation control, smoking prevalence, or left atrial enlargement, confer additional predictive value is essential for tailoring stroke prevention strategies to regional practice realities.

Therefore, this study aims to address an important regional data gap by evaluating demographic characteristics, comorbid conditions, anticoagulation status, and echocardiographic parameters associated with ischemic stroke among patients with non-valvular AF in a tertiary care center in Pakistan.

Objective

The objectives of this study are to determine the frequency and independent predictors of ischemic stroke among patients with non-valvular AF in a tertiary care hospital in Pakistan and to assess whether structural cardiac abnormalities and anticoagulation adequacy provide additional risk stratification beyond conventional clinical risk factors.

## Materials and methods

Study design and setting

This retrospective observational analytic study was conducted at Sir Ganga Ram Hospital, Lahore, Pakistan, from October 2022 to October 2025. The study design was a retrospective cohort analysis using medical records to identify patients with non-valvular AF and determine the occurrence of ischemic stroke during the study period. Patients were followed from the date of AF diagnosis or first hospital encounter within the study timeframe until documented ischemic stroke or the last recorded follow-up visit.

Study population

A total of 225 patients diagnosed with non-valvular AF were included. AF diagnosis was confirmed by electrocardiographic documentation (12-lead ECG or Holter monitoring) recorded in hospital records.

Sample size justification

The sample size of 225 patients was determined by the total number of eligible AF patients meeting the inclusion criteria during the study period. For multivariable logistic regression, the rule of at least 10 outcome events per predictor variable was applied. With 78 stroke events, the study had adequate statistical power to include up to seven predictor variables in the regression model without overfitting.

Inclusion and exclusion criteria

The study included patients aged 18 years or older with a confirmed diagnosis of non-valvular AF. Eligible participants were required to have complete clinical, laboratory, and echocardiographic records, along with documented stroke status during the study period, with ischemic stroke confirmed by CT or MRI. Patients were excluded if they had valvular AF, defined as moderate-to-severe mitral stenosis or the presence of mechanical prosthetic heart valves. Those with a history of intracranial hemorrhage, transient arrhythmias other than AF, or incomplete or missing key clinical records were also excluded from the study.

Outcome definition

The primary outcome was ischemic stroke, confirmed by CT or MRI reports interpreted by certified radiologists. Stroke events included both previously documented ischemic strokes and incident strokes occurring during the study period.

Definition of Anticoagulation Status

“Inadequate anticoagulation” was defined as follows:

For warfarin users: Mean international normalized ratio (INR) <2.0 during follow-up, or documented subtherapeutic INR (<2.0) on ≥2 clinic visits.

For direct oral anticoagulant (DOAC) users: Documented missed doses (≥2 per week), discontinuation (≥48 hours within 30 days) without medical advice, or physician-documented non-adherence in medical records.

Medication adherence was assessed based on clinic documentation, prescription refill records (where available), and physician notes.

Data collection

Data were collected using a standardized structured proforma. Recorded variables included demographic characteristics (age and gender), clinical risk factors such as hypertension, diabetes mellitus, dyslipidemia, and smoking status, and anticoagulation-related variables including the type of anticoagulant used, INR values, and adherence status. Laboratory parameters documented were serum creatinine, brain natriuretic peptide (BNP), and C-reactive protein (CRP). Echocardiographic variables included left atrial enlargement (as defined in echocardiography reports (LA diameter >40 mm)), reduced left ventricular ejection fraction (LVEF <40%), and the presence of left atrial appendage thrombus or spontaneous echo contrast. Patients were subsequently categorized into stroke and non-stroke groups for comparative analysis.

Handling of missing data

Patients with incomplete records for the primary outcome or key exposure variables were excluded at the screening stage. For variables with minor missing values (<5%), complete-case analysis was performed. No imputation methods were applied due to the retrospective design and low proportion of missing data. Analyses involving LAA thrombus/SEC were restricted to patients who underwent transesophageal echocardiography (TEE).

Data analysis

Data were analyzed using IBM SPSS Statistics for Windows, Version 26 (Released 2018; IBM Corp., Armonk, New York, United States). Continuous variables were expressed as mean ± standard deviation (SD) and compared using independent-sample t-tests. Categorical variables were presented as frequencies and percentages and compared using chi-square or Fisher’s exact tests as appropriate. Variables with p <0.10 in univariable analysis and those considered clinically relevant (age, hypertension, anticoagulation status, smoking, left atrial enlargement, LAA thrombus/SEC) were entered into a multivariable logistic regression model using the enter method. Adjusted odds ratios (aORs) with 95% confidence intervals (CIs) were calculated. Multicollinearity was evaluated using the variance inflation factor (VIF). A VIF >5 was considered indicative of significant multicollinearity. No variables exceeded this threshold. Model calibration was assessed using the Hosmer-Lemeshow goodness-of-fit test. Model discrimination was evaluated using the area under the receiver operating characteristic (ROC) curve (AUC). A p-value <0.05 was considered statistically significant. Formal adjustment for multiple comparisons was not applied due to the exploratory nature of the study; however, conclusions are based primarily on adjusted multivariable findings.

## Results

Data were collected from 225 patients with AF. The study population had a mean age of 64.7 ± 10.8 years, and 117 participants (52.0%) were 65 years of age or above. Male patients constituted 134 (59.6%) of the population, while female patients accounted for 91 (40.4%). Hypertension was the most prevalent comorbidity (169; 75.1%), followed by dyslipidemia (131; 58.2%) and diabetes mellitus (112; 49.8%). Smoking was reported in 82 (36.4%) patients. Echocardiographically, 103 (45.8%) patients had left atrial enlargement, and 45 (20.0%) had reduced left ventricular ejection fraction (LVEF <40%). Stroke occurred in 78 patients (34.7%) (Table 1).

**Table 1:** Baseline Characteristics of Patients with Atrial Fibrillation (n = 225) Values are presented as mean ± standard deviation for continuous variables and n (%) for categorical variables. SD, Standard Deviation; LVEF, Left Ventricular Ejection Fraction

Variable	Category/Mean ± SD	n (%)
Age (years)	Mean ± SD	64.7 ± 10.8
Age Groups	<65 years	108 (48.0%)
	≥65 years	117 (52.0%)
Gender	Male	134 (59.6%)
	Female	91 (40.4%)
Hypertension	Present	169 (75.1%)
Diabetes mellitus	Present	112 (49.8%)
Dyslipidemia	Present	131 (58.2%)
Smoking status	Smoker	82 (36.4%)
	Non-smoker	143 (63.6%)
Left atrial enlargement	Present	103 (45.8%)
Reduced LVEF (<40%)	Present	45 (20.0%)
Stroke occurrence	Yes	78 (34.7%)
	No	147 (65.3%)

Patients who experienced stroke were significantly older (68.3 ± 9.7 vs 62.9 ± 11.0 years; p = 0.001). Hypertension was more frequent among stroke patients (64 (82.0%) vs 105 (71.4%); p = 0.03), and smoking prevalence was nearly doubled (38 (48.7%) vs 44 (29.9%); p = 0.005). Left atrial enlargement was strongly associated with stroke (48 (61.5%) vs 50 (34.0%); p < 0.001), as was reduced LVEF (23 (29.4%) vs 21 (14.3%); p = 0.01). TEE was performed in 162 patients (72.0% of the cohort), including 65/78 (83.3%) in the stroke group and 97/147 (66.0%) in the non-stroke group. Among those who underwent TEE, LAA thrombus or SEC was detected in 17/65 (26.2%) stroke patients compared with 9/97 (9.3%) non-stroke patients (p < 0.001) (Table 2).

**Table 2:** Comparison of Clinical and Echocardiographic Features in Stroke vs. Non-stroke Patients p-values were calculated using chi-square tests for categorical variables and independent sample t-tests for continuous variables. Statistical significance was defined as a p-value of less than 0.05. SD, Standard Deviation; LVEF, Left Ventricular Ejection Fraction; LAA, Left Atrial Appendage; SEC, Spontaneous Echo Contrast

Parameter	Stroke (n = 78)	No Stroke (n = 147)	p-value	Mean Difference/Risk Difference
Age (years)	68.3 ± 9.7	62.9 ± 11.0	0.001	5.4 years (95% CI 2.5-8.3)
Male gender	53 (67.9%)	81 (55.1%)	0.04	12.8% (95% CI 0.7-25.0)
Hypertension	64 (82.0%)	105 (71.4%)	0.03	10.6% (95% CI 0.9-20.3)
Diabetes mellitus	44 (56.4%)	68 (46.3%)	0.14	10.1% (95% CI -2.6-22.8)
Dyslipidemia	50 (64.1%)	81 (55.1%)	0.19	9.0% (95% CI -4.1-22.1)
Smoking	38 (48.7%)	44 (29.9%)	0.005	18.8% (95% CI 5.7-31.9)
Left atrial enlargement	48 (61.5%)	50 (34.0%)	<0.001	27.5% (95% CI 13.4-41.6)
Reduced LVEF (<40%)	23 (29.4%)	21 (14.3%)	0.01	15.1% (95% CI 3.1-27.1)
LAA thrombus/SEC (TEE)	17/65 (26.2%)	9/97 (9.3%)	<0.001	16.9% (95% CI 7.8-26.0)

All laboratory measurements were obtained at hospital presentation and therefore reflect post-stroke values. Laboratory findings revealed higher BNP levels in stroke patients (356 ± 128 vs 228 ± 102 pg/mL; p < 0.001) and more frequent CRP elevation (>6 mg/L: 45 (57.6%) vs 43 (29.3%); p < 0.001). Serum creatinine was also higher in stroke patients (1.34 ± 0.42 vs 1.11 ± 0.37 mg/dL; p = 0.002). Of the 118 patients treated with warfarin (52.4% of total cohort), INR values were available for all. Stroke patients receiving warfarin (n = 44) had a significantly lower INR compared with non-stroke warfarin users (n = 74) (1.7 ± 0.6 vs 2.3 ± 0.5; p < 0.001), indicating subtherapeutic anticoagulation (Table 3).

**Table 3:** Laboratory Profile in Stroke vs. Non-stroke Patients Continuous variables are shown as mean ± standard deviation, and categorical variables as n (%). Statistical significance was defined as a p-value of less than 0.05. BNP, Brain Natriuretic Peptide; CRP, C-Reactive Protein; INR, International Normalized Ratio

Laboratory Parameter	Stroke (Mean ± SD / n (%))	No Stroke (Mean ± SD / n (%))	p-value	Mean Difference/Risk Difference
BNP (pg/mL)	356 ± 128	228 ± 102	<0.001	128 pg/mL (95% CI 95-161)
CRP >6 mg/L	45 (57.6%)	43 (29.3%)	<0.001	28.3% (95% CI 14.2-42.4)
Serum creatinine (mg/dL)	1.34 ± 0.42	1.11 ± 0.37	0.002	0.23 mg/dL (95% CI 0.09-0.37)
INR (warfarin users)	1.7 ± 0.6 (n = 44)	2.3 ± 0.5 (n = 74)	<0.001	-0.6 (95% CI -0.8 to -0.4)

Overall, 118 patients (52.4%) received warfarin and 107 (47.6%) received direct oral anticoagulants (DOACs). Inadequate anticoagulation was significantly more frequent in stroke patients (47 (60.3%) vs 41 (27.9%); p < 0.001). Among DOAC users only, discontinuation or missed doses occurred in 21/37 (56.8%) stroke patients versus 18/70 (25.7%) non-stroke patients (p = 0.01). A history of prior transient ischemic attack was more frequently observed in patients who experienced a stroke (18.0% vs 7.5%; p = 0.02), as was poor overall medication adherence 31 (39.7%) vs 36 (24.5%); p = 0.03. The mean CHA₂DS₂-VASc score was significantly higher in the stroke group (4.3 ± 1.2 vs 3.1 ± 1.4; p < 0.001). A score ≥4 was observed in 49 (62.8%) of stroke patients compared with 53 (36.1%) of non-stroke patients (p < 0.001) (Table 4).

**Table 4:** Anticoagulation and Clinical History in Stroke vs. Non-stroke Patients Values are expressed as n (%), with statistical significance determined by chi-square tests. Statistical significance was defined as a p-value of less than 0.05. DOAC, Direct Oral Anticoagulant

Parameter	Stroke (n = 78)	No Stroke (n = 147)	p-value	Risk Difference/Mean Difference
Inadequate anticoagulation	47 (60.3%)	41 (27.9%)	<0.001	32.4% (95% CI 18.1-46.7)
DOAC discontinuation/missed doses	21/37 (56.8%)	18/70 (25.7%)	0.01	31.1% (95% CI 12.2-50.0)
Prior transient ischemic attack	14 (18.0%)	11 (7.5%)	0.02	10.5% (95% CI 1.6-19.4)
Poor adherence to medications	31 (39.7%)	36 (24.5%)	0.03	15.2% (95% CI 1.8-28.6)
Mean CHA₂DS₂-VASc score (mean ± SD)	4.3 ± 1.2	3.1 ± 1.4	<0.001	Mean difference: 1.2 (95% CI 0.8-1.6)
High-risk CHA₂DS₂-VASc score (≥4)	49 (62.8%)	53 (36.1%)	<0.001	26.7% (95% CI 12.7-40.7)

Multivariable logistic regression identified several independent predictors of stroke. A backward stepwise elimination strategy was applied. Patients aged 65 years or older had over twice the likelihood of experiencing a stroke (aOR 2.41; 95% CI 1.32-4.39; p = 0.004). Hypertension nearly doubled stroke risk (aOR 1.98; 95% CI 1.05-3.72; p = 0.03). Left atrial enlargement (aOR 2.89; p = 0.001) and LAA thrombus/SEC (aOR 3.72; p < 0.001) were strong predictors. Inadequate anticoagulation increased stroke odds over threefold (aOR 3.15; p < 0.001), while smoking was also an independent predictor (aOR 1.84; p = 0.03) (Table 5).

**Table 5:** Multivariable Logistic Regression for Predictors of Stroke Adjusted odds ratios (aORs) with 95% confidence intervals (CIs) are presented. Statistical significance was defined as a p-value of less than 0.05. Variables tested but excluded: Reduced LVEF, BNP, CRP, serum creatinine, male sex, prior TIA. Events-per-variable ratio: 13; no multicollinearity observed (VIF <2.5). Model discrimination C-statistic = 0.81 (95% CI 0.75-0.87); Hosmer-Lemeshow p = 0.47. A sensitivity analysis excluding anticoagulation-related variables yielded consistent associations for age ≥65 years, left atrial enlargement, and LAA thrombus/SEC, with <10% change in effect estimates. LAA, Left Atrial Appendage; SEC, Spontaneous Echo Contrast

Variable	aOR	95% CI	p-value
Age ≥65 years	2.41	1.32-4.39	0.004
Hypertension	1.98	1.05-3.72	0.03
Left atrial enlargement	2.89	1.56-5.36	0.001
LAA thrombus/SEC	3.72	1.72-8.03	<0.001
Inadequate anticoagulation	3.15	1.72-5.77	<0.001
Smoking	1.84	1.05-3.23	0.03

## Discussion

This retrospective study evaluated predictors of ischemic stroke in 225 patients with AF, among whom 78 (34.7%) experienced thromboembolic events. Stroke events in this cohort were prevalent at the time of presentation or documented during hospital follow-up; the study design does not allow causal inference for incident events. Nevertheless, the analysis provides quantifiable associations between patient characteristics, structural cardiac changes, anticoagulation adequacy, and stroke risk.

Age ≥65 years independently increased stroke risk over twofold (aOR 2.41; 95% CI 1.32-4.39; p = 0.004), consistent with prior studies and reflected in the age component of CHA₂DS₂-VASc [14]. Hypertension was similarly a significant independent predictor (aOR 1.98; 95% CI 1.05-3.72; p = 0.03), supporting its established contribution to atrial remodeling and thromboembolic risk [15]. These findings reinforce the incremental value of traditional clinical risk factors in the local Pakistani population, where hypertension prevalence is high.

Left atrial enlargement nearly tripled stroke odds (aOR 2.89; 95% CI 1.56-5.36; p = 0.001), while LAA thrombus or SEC was the strongest predictor (aOR 3.72; 95% CI 1.72-8.03; p < 0.001) [16]. These structural markers capture atrial remodeling and stasis beyond standard CHA₂DS₂-VASc parameters, highlighting the value of echocardiographic assessment, particularly in patients with intermediate risk scores. However, only a subset of patients underwent TEE, limiting generalizability. In resource-limited settings without routine TEE, identification of left atrial enlargement on transthoracic echocardiography may serve as a pragmatic surrogate.

BNP and CRP were elevated among stroke patients, suggesting prothrombotic hemodynamics and systemic inflammation [17,18]. While these markers were not included in the final regression model due to collinearity and missing values, their elevated levels may reflect residual risk. Reverse causality is possible, as BNP, CRP, and creatinine could be influenced by acute stroke or concomitant heart failure, rather than serving solely as independent predictors.

Inadequate anticoagulation markedly increased stroke risk (aOR 3.15; 95% CI 1.72-5.77; p < 0.001) [19]. Subtherapeutic INR in warfarin users (mean 1.7 ± 0.6 vs 2.3 ± 0.5; p < 0.001) and missed or delayed DOAC doses were key contributors. When stratified, warfarin users with subtherapeutic INR had a higher proportion of stroke than adherent DOAC users, though the study was underpowered for formal warfarin vs DOAC outcome comparisons. These findings reinforce the importance of routine monitoring, adherence assessment, and individualized anticoagulation strategies in clinical practice.

Smoking independently predicted stroke (aOR 1.84; 95% CI 1.05-3.23; p = 0.03), likely through endothelial dysfunction, inflammation, and platelet activation. Other comorbidities, diabetes, dyslipidemia, and reduced LVEF, were more prevalent in stroke patients but were not statistically significant in multivariable analysis, suggesting limited incremental predictive value in this cohort [20,21].

CHA₂DS₂-VASc remains a validated and widely used tool, but our findings suggest that incorporating structural (left atrial enlargement, LAA thrombus) and therapeutic factors (anticoagulation adequacy) can refine risk stratification in the local context [10-12]. For example, patients with CHA₂DS₂-VASc scores in the intermediate range may be reclassified as high-risk if imaging or adherence data reveal additional risk.

The retrospective design limits causal inference and may introduce residual confounding. Missing data for BNP, CRP, creatinine, and echocardiographic parameters were handled by listwise deletion, which could bias estimates. Stroke temporality relative to lab measurements was not always available, raising potential reverse causality. Anticoagulation compliance was assessed through chart review and patient reporting, potentially underestimating nonadherence. Single-center data may limit generalizability to broader populations with differing healthcare access, anticoagulation practices, and imaging availability.

In settings where TEE is not routinely available, transthoracic echocardiographic detection of left atrial enlargement may serve as a practical risk marker. Ensuring therapeutic anticoagulation, emphasizing adherence, and addressing modifiable lifestyle factors such as smoking are critical for stroke prevention. Our study highlights the potential for locally adapted risk stratification combining conventional CHA₂DS₂-VASc parameters with structural and therapeutic variables to guide personalized management strategies.

## Conclusions

In this retrospective cohort of patients with AF, stroke risk was independently associated with older age, hypertension, left atrial enlargement, LAA thrombus or SEC, inadequate anticoagulation, and smoking. These findings emphasize the importance of refining risk stratification beyond conventional tools such as CHA₂DS₂-VASc by incorporating structural cardiac markers and anticoagulation quality.

Ensuring therapeutic anticoagulation and adherence, alongside identification of high-risk structural features, may improve stroke prevention and guide targeted clinical interventions. Prospective multicenter studies are warranted to validate these predictors and assess their generalizability across broader patient populations and different healthcare settings.
